# The therapeutic efficacy of Huashi Baidu Formula combined with antiviral drugs in the treatment of COVID-19: A protocol for systematic review and meta-analysis

**DOI:** 10.1097/MD.0000000000022715

**Published:** 2020-10-16

**Authors:** Lizhu Han, Yunlan Wang, Kunxia Hu, Zhishu Tang, Xiao Song

**Affiliations:** College of Pharmacy, Shaanxi University of Chinese Medicine, Xianyang, China.

**Keywords:** antiviral drugs, COVID-19, Huashi Baidu Formula, meta-analysis, protocol, systematic review

## Abstract

**Background:**

The Corona Virus Disease 2019 (COVID-19) is a new acute respiratory infectious disease that has become a major global public health event. In China, the combination of Huashi Baidu Formula (HBF) and antiviral drugs is used in the clinical treatment of severe patients with new coronary pneumonia, but there is still a lack of evidence-based medical evaluation.

**Methods:**

We search for research in PubMed/MEDLINE, China National Knowledge Infrastructure (CNKI), Wanfang Database, VIP Database, Cochrane Library, Embase, China Biomedical Database (CBM), and Chinese Science Citation Database (CSCD). For “Huashi Baidu Formula” and “COVID-19,” we screened suitable articles without language restrictions on keywords, and recorded and analyzed the screened literature with RevMan 5.3 and STATA 14.2 software.

**Results:**

This systematic review and meta-analysis will evaluate the efficacy and safety of HBF combined with antiviral drugs in the treatment of COVID-19, and provide the rationality of clinical drug application.

**Conclusion:**

Our findings will provide references for clinical diagnosis and treatment and guidance programs for COVID-19.

**INPLASY registration number:**

INPLASY202080098.

## 1 Introduction

Since the outbreak of the corona virus disease (COVID-19) in December 2019, it has spread rapidly in various countries around the world due to its long incubation period, strong contagiousness and pathogenicity, and general susceptibility to various groups of people. According to statistics from the World Health Organization, as of 5 September 2020, there were 26,468,031 confirmed cases worldwide, with a total of 871,166 deaths, and 216 countries or regions with cases.^[1]^ The COVID-19 pandemic has a great negative impact on human health and world economic and social development.

China has made significant contributions to the detection and treatment of COVID-19. Among them, traditional Chinese medicine (TCM) has accumulated rich experience and practical basis in the long-term epidemic prevention and treatment. HBF was summarized by the medical team of the Chinese Academy of Chinese Medical Sciences in Wuhan Jinyintan Hospital based on clinical experience. In China's Diagnosis and treatment of corona virus disease-19 (trial seventh and eighth editions), HBF is listed as the recommended medicine for patients with severe COVID-19.^[2,3]^ HBF is mainly for patients with clinical manifestations of fever, flushing, cough, yellow and sticky sputum, or blood in sputum, shortness of breath, fatigue, dry mouth, sticky mouth, nausea, inability to eat, poor stool, and short red urine. The pharmacological research results of HBF and its clinical practice verified each other, indicating the effectiveness of HBF in the treatment of COVID-19.^[4,5]^ However, there is no evidence-based medicine research on HBF combined with antiviral drugs. Therefore, we have proposed a systematic evaluation program to evaluate the efficacy and safety of HBF combined with antiviral drugs for COVID-19.

## 2 Materials and methods

Our protocol has been registered on the International Platform of Registered Systematic Review and Meta-Analysis Protocols (INPLASY). The registration number was INPLASY202080098 (DOI: 10.37766/inplasy2020.8.0098). We strictly abide by Preferred Reporting Items for Systematic review and Meta-Analysis Protocols (PRISMA-P) guidelines.^[6]^

### 2.1 Data source and retrieval strategy

**Figure 1:**
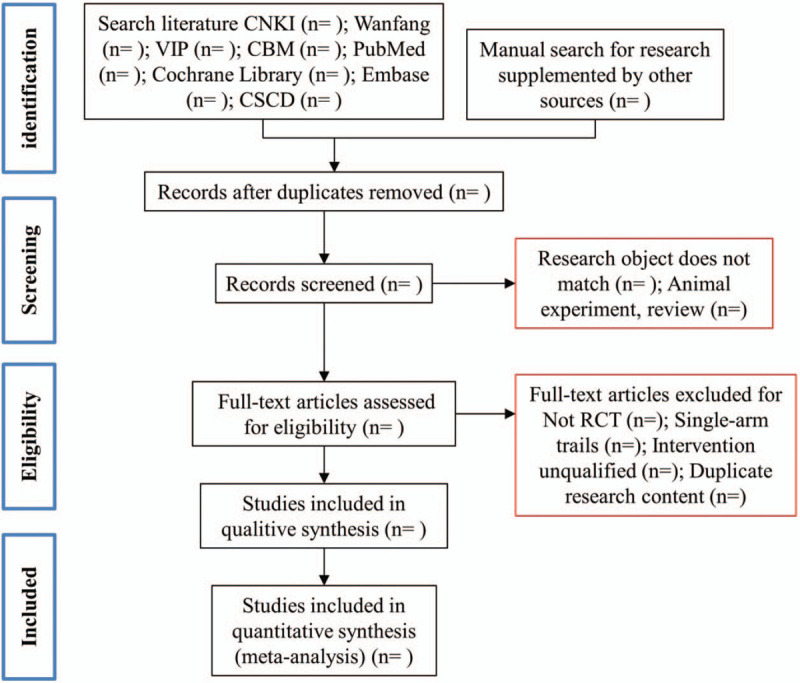
Flow chart for screening qualified studies.

We conduct literature searches in PubMed, the China National Knowledge Infrastructure (CNKI), Wanfang database, VIPdatabase, the Cochrane Library, Embase, the Chinese Biomedical Database (CBM) and the Chinese Science Citation Database (CSCD). Among them, “Huashi Baidu Formula,” “Huashi Baidu Decoction,” “COVID-19,” “Corona Virus Disease 2019,” “severe pneumonia,” “randomized controlled trial” as the theme or keywords perform a search. There are no language restrictions on the subject and keyword search. The results of the electronic database search are supplemented by manual search research. Two researchers independently assessed the quality of the included studies, excluded low-quality studies, and used different statistical models for data analysis. The complete screening process is shown in Figure 1.

### 2.2 Inclusion and exclusion criteria

Included in clinical randomized controlled trials (RCTs) of HBF in the treatment of COVID-19, whether blinded or not. There is no language limitation. Retrieving the studies from December 2019 to the present, the research subjects are not limited in gender and age, and meet the diagnostic criteria according to the “ Diagnosis and treatment of corona virus disease-19” issued by the National Health Commission of China.^[7]^ The experimental group was treated with HBF combined with antiviral drugs to treat COVID-19, and the control group was treated with antiviral drugs alone or other drugs.

The diagnostic criteria were as follows:

1. The main manifestations of patients are fever, dry cough, and fatigue. Critically ill patients present with dyspnea or hypoxemia; shortness of breath, RR ≥ 30 times/min.
2. The patient has decreased lymphocyte count, elevated C-reactive protein (CRP) and erythrocyte sedimentation rate, and normal procalcitonin.^[8]^
3. The nucleic acid of the Corona Virus Disease 2019 is detected in samples such as nasopharyngeal swabs, sputum and other lower respiratory secretions, blood, feces, and urine during the pathogenic examination. The serum Immunoglobulin M (IgM) antibody and Immunoglobulin (IgG) antibody were positive.^[9]^

The exclusion criteria were as follows: studies that could not accurately extract data or data were missing; for studies published repeatedly, the study with the most comprehensive data was selected.

### 2.3 Quality assessment of included studies

Two experimenters sorted out the research obtained from the screening into a table, and the extracted information was:

1. Basic characteristics of the included literature (author, publication time);
2. Basic information of the research object (number of samples in each group, age, ratio of male to female, course of disease, adverse event [AE]);
3. Detailed content of intervention measures (drugs and time used in treatment);
4. Outcome indicators and final data results.

The extracted data were assessed for Cochrane bias risk, and Low (Low bias), Unclear (Unclear bias), High (High bias) were judged respectively on the generation of random sequences, allocation concealment, implementation of blind method, incomplete outcome data, selective reporting outcomes, and other biases.^[10]^ Seek third-party opinions when there are differences or inability to judge other biased research screening and quality evaluation.

### 2.4 Data analysis

Review Manager 5.3 and STATA 14.2 software were used for the meta-statistical analysis of the included studies. The total effective rate (TER), the disappearance rate of main clinical features (DROMCF) and the disappearance rate of minor symptoms (DROMS) are regarded as dichotomous variables. It is expressed in odds ratio (OR) and described with 95% confidence interval (95% CI).^[11]^ The number of proinflammatory cytokines, WBC, LYM, CRP, IgG, and IgM are continuous variables, using weighted mean difference (WMD) or standardized mean difference (SMD) as the effect index, and 95% CI for description. The *Q* test was used for analysis, combined with *I*^2^ to quantitatively determine the degree of heterogeneity. A fixed-effects model was used to analyze data with low heterogeneity (*P* ≥ .1 and *I*^2^ ≤ 50%) and data with high heterogeneity (*P* < .1 or *I*^2^ > 50%) was estimated using random-effects model. Potential publication bias was revealed by funnel plots.^[12]^

## 3 Discussion

TCM has a unique theoretical and practical basis in fighting against the plague. It has achieved good curative effect in the fight against covid-19 in China. Chinese medicine can effectively reduce the rate of weight conversion according to the patient's different constitution. Especially in the early intervention, it can effectively reduce the development of light and common type from heavy to severe and from severe to critical. The combined application of TCM and antiviral drugs can quickly improve symptoms such as fever, cough and fatigue, increase the rate of nucleic acid conversion, increase the cure rate, and reduce the mortality rate. The clinical research, network pharmacology and molecular docking studies of HBF show that it has good curative effect in the treatment of severe COVID-19 patients.^[13]^ This study provides an evaluation of the efficacy and safety of HBF in the treatment of COVID-19, and provides a basis for clinical diagnosis and treatment.

## Author contributions

**Conceptualization:** Xiao Song.

**Data curation:** Lizhu Han, Yunlan Wang, Zhishu Tang.

**Formal analysis:** Kunxia Hu.

**Funding acquisition:** Yunlan Wang.

**Project administration:** Xiao Song.

**Software:** Kunxia Hu.

**Supervision:** Zhishu Tang.

**Visualization:** Kunxia Hu.

**Writing – original draft:** Lizhu Han.

**Writing – review & editing:** Xiao Song.
